# Transfusion of blood products to neonatal and pediatric extracorporeal membrane oxygenation patients: A prospective, observational study

**DOI:** 10.1111/trf.70154

**Published:** 2026-06-18

**Authors:** Rebecca Barton, Suelyn Van Den Helm, Hui Ping Yaw, Chantal Attard, Graeme MacLaren, Vera Ignjatovic, Natasha Letunica, Vasiliki Karlaftis, Tengyi Cai, Amy Johansen, Derek Best, Roberto Chiletti, Yves d'Udekem, Jo McKittrick, Warwick Butt, Paul Monagle

**Affiliations:** ^1^ Haematology Research Murdoch Children's Research Institute Melbourne Australia; ^2^ Department of Paediatrics The University of Melbourne Melbourne Australia; ^3^ Clinical Haematology The Royal Children's Hospital Melbourne Australia; ^4^ Haematology & Blood Bank KK Women's & Children's Hospital Singapore Singapore; ^5^ Shanghai Immune Therapy Institute Shanghai Jia Tong University School of Medicine Shanghai China; ^6^ Cardiothoracic Intensive Care Unit National University Health System Singapore Singapore; ^7^ John Hopkins All Children's Institute for Clinical and Translational Research St Petersburg USA; ^8^ Department of Paediatrics, School of Medicine John Hopkins University Baltimore USA; ^9^ Intensive Care The Royal Children's Hospital Melbourne Australia; ^10^ Paediatric Intensive Care Research Group Murdoch Children's Research Institute Melbourne Australia; ^11^ Cardiovascular Surgery Children's National Heart Institute Washington USA; ^12^ Department of Critical Care The University of Melbourne Melbourne Australia

## Abstract

**Study Question:**

To describe the blood product utilization during neonatal and pediatric extracorporeal membrane oxygenation (ECMO) and to determine whether its usage correlates with clinical outcomes of bleeding, thrombosis or mortality.

**Design:**

Prospective observational study of neonatal and pediatric ECMO patients from September 2016, until January 2022.

**Setting:**

Tertiary center for pediatric and neonatal ECMO.

**Patients:**

One hundred and ten runs of ECMO, nine patients had two runs of ECMO during the same hospital admission.

**Results:**

All patients received at least one red blood cell (RBC) transfusion. RBCs, as well as fresh frozen plasma (FFP), platelets and cryoprecipitate transfusion, as well as total donor exposure did not predict increased mortality in ECMO patients. Increased volume of platelets was associated with patients who bled, but not those who had a thrombosis or died.

**Discussion:**

This prospective cohort study failed to demonstrate that the volume of red blood cells, FFP and platelets or total donor exposure was predictive of increased mortality. Of particular importance is the lack of increased volume of red blood cells transfused to patients who experienced major bleeding events compared to those who did not, suggesting that the trigger for transfusions may not be based on the clinical status of bleeding and resultant Hb in isolation.

**Abbreviations**
ACT: activated clotting time
ANOVA: analysis of variance
APTT: activated partial thromboplastin time
CNS: central nervous system
CPB: cardiopulmonary bypass
crSO2: cerebral regional tissue oxygenation
CVVH: continuous veno‐venous hemofiltration
ECLS: extracorporeal life support
ECMO: extracorporeal membrane oxygenation
ECPR: extracorporeal cardiopulmonary resuscitation
ELSO: Extracorporeal Life Support Organization
EMR: electronic medical record
FFP: fresh frozen plasma
g/dL: grams per deciliter
Hb: hemoglobin
IQR: interquartile range
LMS: lab management system
PICU: pediatric intensive care unit
Plt: platelet
RBC: red blood cell
RCH: Royal Children's Hospital
RCT: randomized control trial
UFH: unfractionated heparin
VA: venoarterial
VV: venovenous

## 1 INTRODUCTION

Extracorporeal membrane oxygenation (ECMO) is a modified form of cardiopulmonary bypass used in pediatric and neonatal patients to provide mechanical circulatory or respiratory support for refractory heart or lung failure. Current management of patients on ECMO utilizes systemic anticoagulation to prevent patient and circuit‐based thrombosis. Despite significant advances, hemorrhage and thrombosis remain the most common cause of morbidity and mortality in these patients.^1^ Depending upon the underlying patient pathophysiology, the sequence of events that leads to the initiation of ECMO can vary, with some patients electively commenced following cardiac surgery, or infective illness, or considered in an emergent setting for cardiac arrest (ECPR). Prior to the commencement of, and during, ECMO, blood products are transfused to the patient to assist with control of hemostasis, hypovolemic, hypoxia, and anemia.

Transfusion of blood products may include red blood cells (RBCs), platelets, fresh frozen plasma (FFP) and cryoprecipitate, with current research demonstrating mixed results with regards to specific products and their related clinical outcomes. Some studies have suggested transfusion of blood products is associated with increased mortality in both the critical care and ECMO setting.^2^, ^3^, ^4^, ^5^, ^6^, ^7^, ^8^, ^9^ Contrary to this, two randomized controlled trials (RCTs) within critical care demonstrated no significant difference in mortality between liberal and restrictive transfusion protocols.^10^, ^11^ With a lack of ECMO‐specific RCTs, the Extracorporeal Life Support Organization (ELSO) have recommended that to achieve optimal oxygenation, selected patients may require transfusion of RBCs to above previously accepted hemoglobin (Hb) thresholds.^12^

We present a single center prospective cohort of pediatric and neonatal ECMO patients with complete data on blood product usage, the volume and type of blood products, and donor exposure during ECMO. The purpose is to comprehensively describe a prospective cohort, in an attempt to increase the understanding of blood product usage and determine if there is a relationship between the transfusion of blood products and outcomes of mortality, thrombosis and major bleeding, utilizing a priori definitions.

## 2 MATERIALS AND METHODS

Neonatal and pediatric ECMO patients were recruited at The Royal Children's Hospital (RCH), Melbourne, Australia, from September 2016 to January 2022. This study received ethics approval by The Royal Children's Hospital Ethics in Human Research Committee (HREC number 35252).

Written informed consent was obtained from the parent or legal guardian of all enrolled participants within 48 h of commencing ECMO as per the ethics approval for this study.

For the full ECMO study protocol please refer to the previously published by Van Den Helm et al.^13^

### 2.1 Inclusion criteria

All neonatal and pediatric patients in The Royal Children's Hospital Pediatric Intensive Care Unit (PICU) who were cannulated onto either venovenous (VV) or venoarterial (VA) ECMO.

### 2.2 Exclusion criteria

Patients with a history of a hematological disorder, gestational age less than 34 weeks, projected ECMO duration of less than 48 h, those who were cannulated at an external hospital to RCH, or whose parent or legal guardian did not speak or read English were excluded from the study. In addition, following discussion with the treating physician a number of patients were not approached due to exceptional psychosocial factors and involvement would cause significant distress within the family.

### 2.3 Clinical data

The patient electronic medical record (EMR) was used to obtain information relating to; age, sex, weight, height, past medical history, indication for ECMO, ECMO details (mode, cannulation type, additional cannulas, anticoagulation administration, transfusion of blood products), PICU and hospital admission duration, bleeding and thrombotic events, and survival to hospital discharge.

### 2.4 Bleeding and thrombosis a priori definitions

The definition of major bleeding was established using a combination of the RCH Extracorporeal Life Support (ECLS) protocol and ELSO published data registry definitions, to include blood loss of >4 mL/kg/h for greater than 4 h, or site‐specific bleeding such as intracranial or pulmonary hemorrhage confirmed on imaging, gastrointestinal bleeding requiring endoscopic or surgical intervention, and surgical site bleeding requiring re‐operation.^14^

Thrombosis was defined as radiologically proven thrombosis (venous or arterial) requiring acute intervention or formal anticoagulation beyond the period of ECMO, as well as radiologically confirmed central nervous system (CNS) embolic stroke or ECMO circuit thrombosis requiring a circuit change. A circuit change is indicated for oxygenator failure, diffuse clot on arterial side, hemolysis secondary to clot burden in the circuit or oxygenator/pump, pump failure, or elective changes in theater during transitions between ECMO and cardiopulmonary bypass (CPB). The diagnosis and indications for thrombosis and circuit changes were confirmed by PICU and hematology staff. Routine surveillance imaging was not performed.

### 2.5 Blood product transfusion

Transfusion of blood products was according to RCH ECLS protocol targets and are: Hb ≥80–100 g/L, platelets ≥801–100 × 10^9^/L and fibrinogen >1.5 g/L. However, there was no restriction on individual clinician decision making. Documentation of all transfused blood products was obtained from the EMR, and due to the exclusion of this input method during massive transfusion events, additional transfusion product documentation was obtained from the Medipath (Lab Management System—LMS) system. The product was then cross‐checked against the product ID to ensure transfusion to the correct patient.

Transfused blood products were calculated in ml/kg given the variation in weight and transfusion requirements of patients. All blood products were collected, processed and stored by the Australian Red Cross LifeBlood® according to nationally produced guidelines.^15^ All red cell products were leukodepleted, and both platelets and red blood cells were irradiated, except in massive transfusion events where demand exceeded irradiation capacity. Platelets were either whole blood derived or apheresed, depending upon availability and underlying patient pathophysiology. The majority of FFP and cryoprecipitate was whole blood derived. Transfusion of all blood products was standardized to mL/kg/day to allow for direct comparison between patients and outcomes. RBC transfusions were separated into those that were ordered for priming or replacement of circuits (ECMO), cardiopulmonary bypass (CPB) and continuous veno‐venous hemofiltration (CVVH), referred to generally as a circuit prime, and those that were not. Donor exposure refers to the number of different blood donors each patient was exposed to as a result of blood product transfusion.

### 2.6 Anticoagulation protocol

All anticoagulation was guided by the RCH ECLS protocol and included a loading dose (50–100 Units/kg) of unfractionated heparin (UFH) administered prior to cannulation, followed by a continuous infusion of UFH (10–40 Units/kg/h) for the duration of ECMO. The continuous infusion was guided by a combination of anticoagulation tests (APTT, Anti‐Xa, and ACT). Epoprostenol (5 ng/kg/min) and nitric oxide were also administered.

### 2.7 Statistical analysis

Data was summarized with descriptive statistics, including means and standard deviations or medians and interquartile ranges (IQRs) for continuous data, or with frequencies and percentages for categorical data. The incidence of thrombosis and bleeding was calculated as a percentage of all ECMO runs. Univariate analysis between volume of transfused blood products and clinical outcomes of thrombosis, bleeding, and mortality was performed using T sample *T*‐tests. One‐way ANOVAs (where equal variances were not assumed) and Games–Howell pairwise comparisons were performed with respect to age categories and primary ECMO indications. Co‐variate and multivariate analysis of both categorical and continuous variables was performed with general linear regression and binary logistic regression, respectively. All data was analyzed with Minitab® version 21.4.2 (Minitab LLC), and all *p*‐values of less than .05 were considered statistically significant.

## 3 RESULTS

Patient characteristics are summarized in Table 1 with additional details of red cell transfusions, platelets, FFP, and cryoprecipitate shown in Table 2.

**TABLE 1:** Patient characteristics.

Patient characteristics	*n* = 110
Age (months), median (IQR)	1.81 (0.16–25.95)
Range (months)	0–18
Age category, *n* (%)	
Neonate (0–30 days of life)	48 (44%)
Infant (31 days–1 year)	22 (22%)
Child (>1–18 years)	40 (36%)
Sex, *n* (%)	
Female	55 (50%)
Male	55 (50%)
Weight (kg), median (IQR)	4 (3–10)
Range (kg)	2–63
Indication for ECMO, *n* (%)	
Cardiac	82 (75%)
Respiratory	19 (17%)
Other	9 (8%)
Cardiopulmonary bypass (CPB), *n* (%)	
Yes	57 (52%)
No	53 (48%)
Mode of ECMO, *n* (%)	
Venovenous (VV)	10 (9%)
Venoarterial (VA)	100 (91%)
Type of cannulation, *n* (%)	
Peripheral	37 (34%)
Central	73 (66%)
Duration of ECMO (d), median (IQR)	5 (3–8)
Range (days)	1–90
PICU length of stay (day), median (IQR)	21 (11–35)
Hospital length of stay (day), median (IQR)	37 (21–81)
Survival to discharge	
Age categories, *n* (%)	
Neonate	
Yes/no	27/21 (56%/44%)
Infant	
Yes/no	19/3 (86%/14%)
Child	
Yes/no	31/9 (78%/22%)
All ages, *n* (%)	
Yes/no	77/33 (70%/30%)
Clinical outcomes	
At least one major bleed ONLY	38 (35%)
At least one thrombotic event ONLY	9 (8%)
Both major bleed and thrombotic events	10 (9%)

**TABLE 2:** Comparison of RBC volumes when adjusted for age, weight and duration of ECMO, as well as other adjusted blood products.

Variable	Mean	SE mean	SD	Minimum	Median	Maximum
Comparison of red blood cell (RBC) volumes						
Total RBC vol (mL)	1278	179	1873	200	847	18,335
RBC (mL/kg)	205.1	17.2	180.0	4.1	166.1	967.7
Daily RBC (mL/kg)	41.2	3.6	36.9	0.5	34.9	232.7
Other transfused blood products						
Daily Plt (mL/kg)	12.2	0.9	9.0	0	11.2	44.6
Daily FFP (mL/kg)	20.0	2.0	21.1	0	15.2	93.9
Daily Cryo (mL/kg)	4.5	0.5	5.2	0	2.9	25.0

Abbreviations: Cryo, cryoprecipitate; FFP, fresh frozen plasma; PLT, platelets; RBC, red blood cells.

The total duration of ECMO run (days) grouped into Primary ECMO indication (cardiac, other, or respiratory), as well as total volume of RBC transfused to each respective group is shown in Figures S1 and S2.

Figure 1 displays the association between transfusion of different blood products, the ECMO mode (VV or VA), and the cannulation type (peripheral or central). VA ECMO was statistically associated with a higher volume of all blood products except cryoprecipitate when compared to VV ECMO. Central cannulation was also statistically associated with a higher volume of all blood products when compared to peripheral cannulation.

**FIGURE 1:**
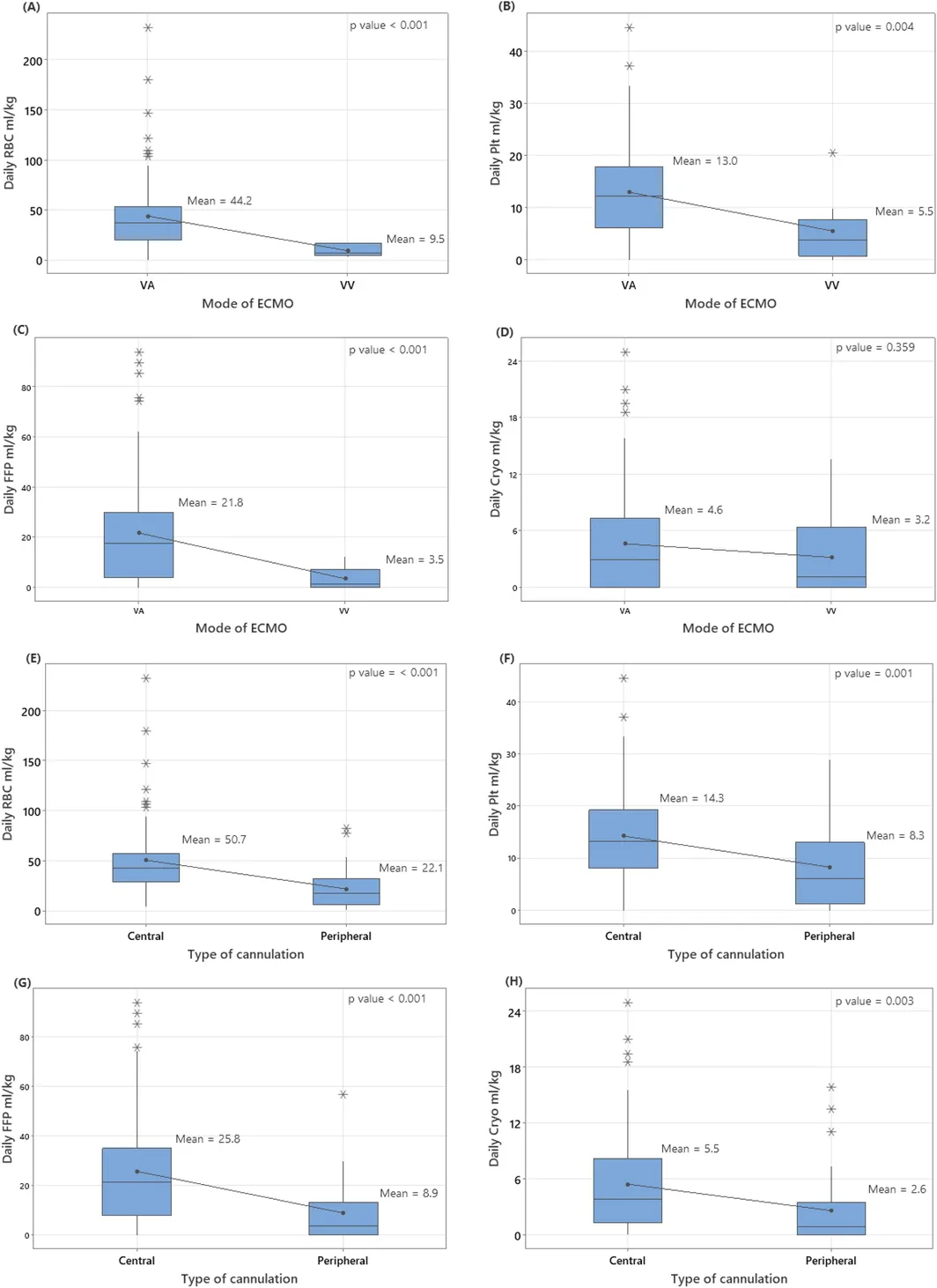
Panels A–D illustrate the relationship between daily volumes of blood products (RBC, Plt, FFP, and Cryo) and ECMO modality. Panels E–H demonstrate the relationship between daily volumes of these blood products and cannulation type. Statistical analyses were conducted using the two‐sample *t*‐test.

Figures 2 and 3 demonstrate the relationship between daily product transfusions and clinical outcomes of mortality, major bleeding, and thrombosis. There was no statistically significant difference in the volume of these blood products and survival to discharge or thrombosis. Increased volumes of platelets were associated with major bleeding.

**FIGURE 2:**
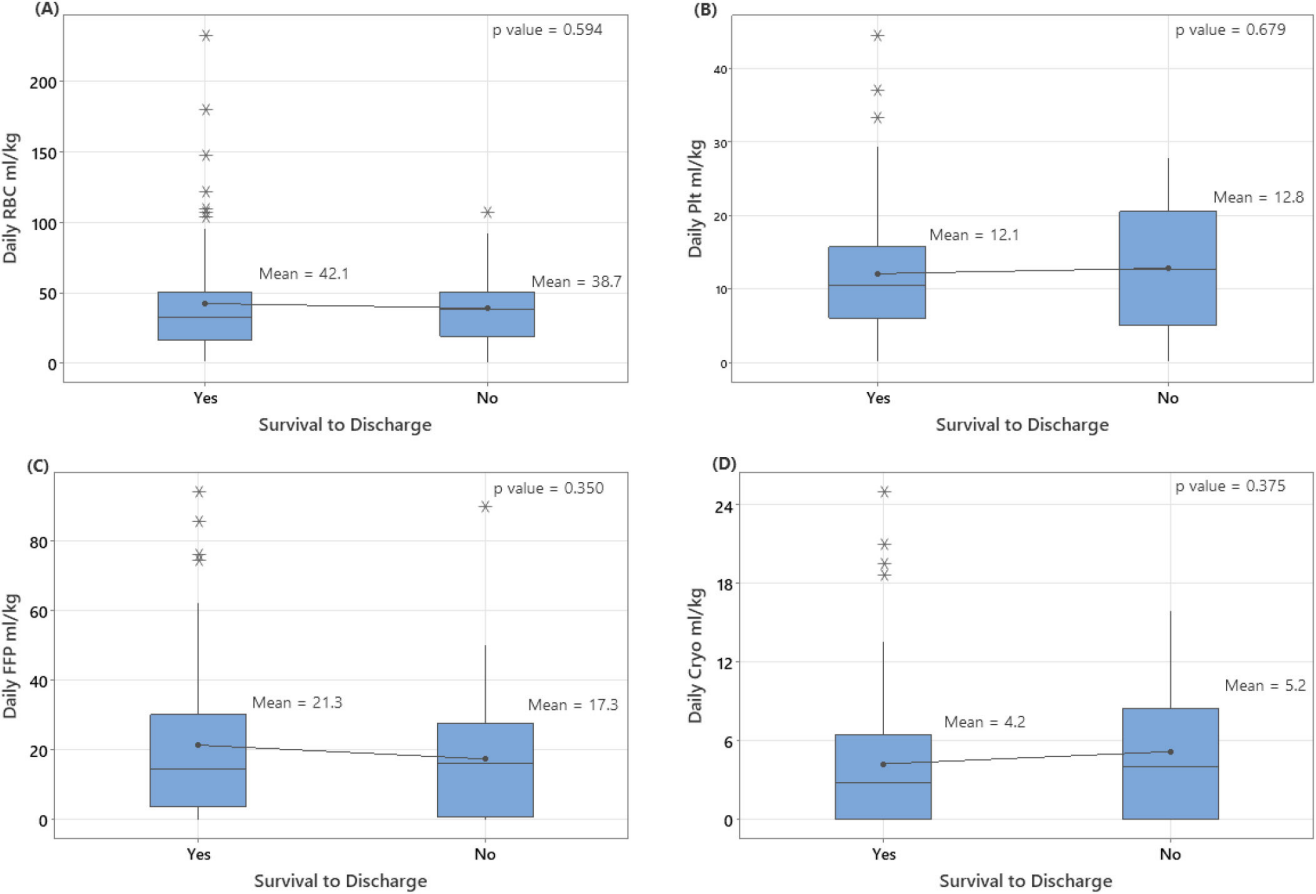
Panels A–D illustrate the relationship between daily product transfusions and survival to discharge. Panel A: RBC; B: Plt; C: FFP; D: Cryo.

**FIGURE 3:**
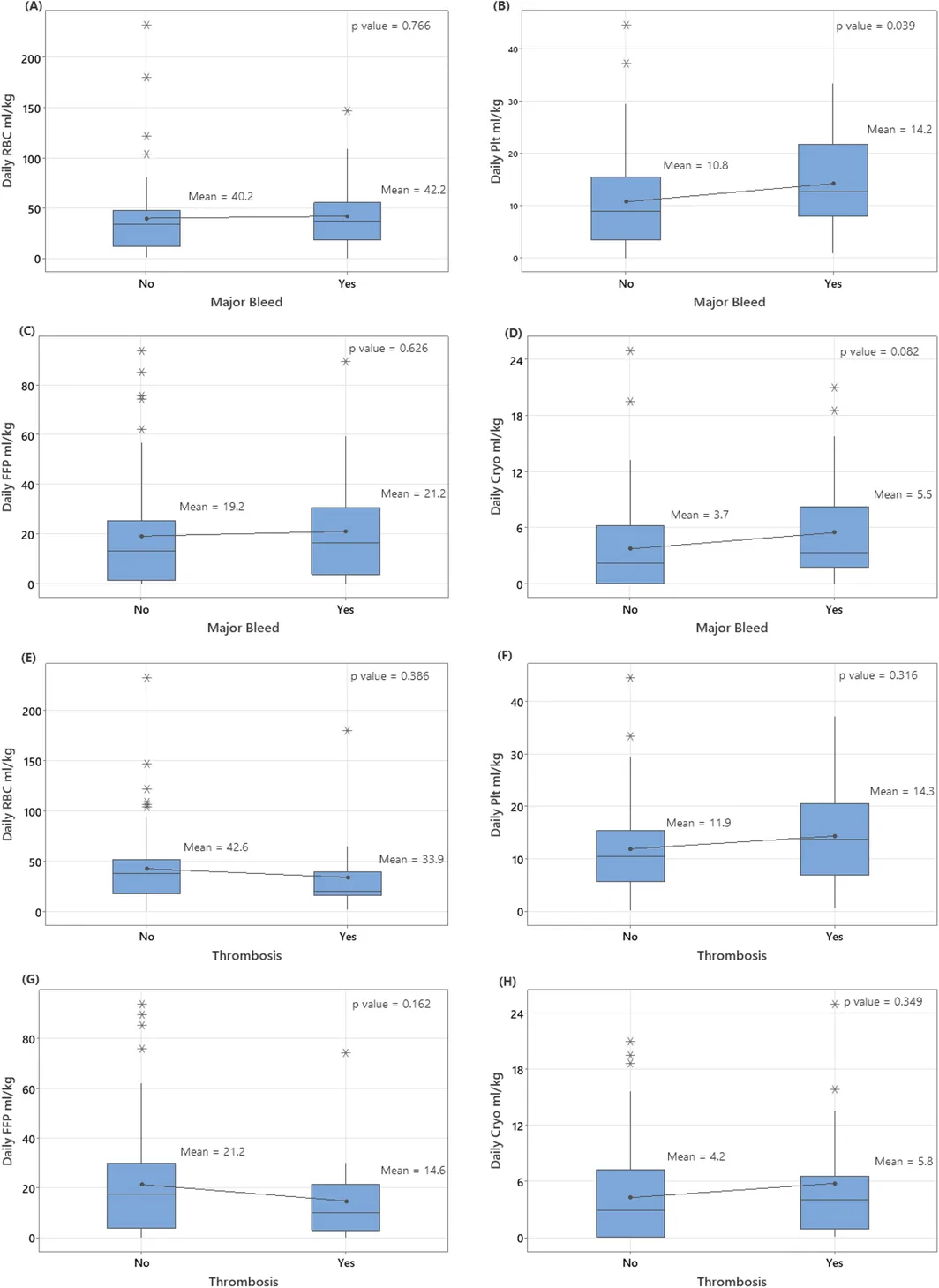
Panels A–D illustrate the relationship between daily product transfusions and major bleeding. Panel A: RBC; B: Plt; C: FFP; D: Cryo. Panels E–H illustrate the relationship between daily product transfusions and thrombosis. Panel E:– RBC; F: Plt; G: FFP; H: Cryo.

Figure 4 presents an analysis of daily product transfusion in patients who required ECMO following CPB  and those who did not. Transfusion rates for all blood products, excluding platelets, were statistically higher among patients who required ECMO after CPB, regardless of whether the circuit prime was included.

**FIGURE 4:**
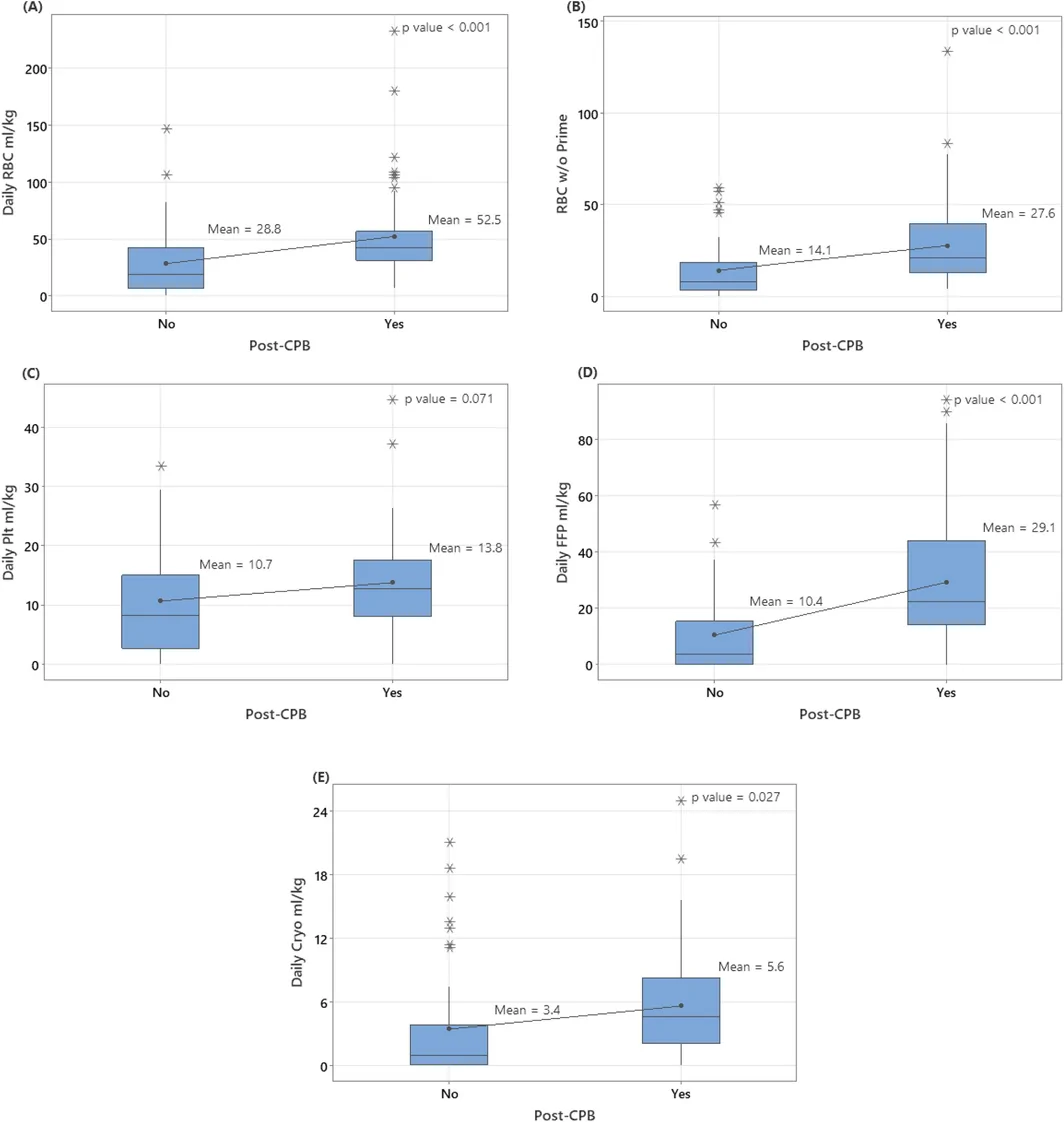
Panels A–E illustrate the relationship between daily product transfusions and patients who required ECMO post‐CPB and those who did not, with and without circuit prime. Panel A: RBC; B: RBC w/o prime; C: Plt; D: FFP; E: Cryo.

The donor exposure relationship with mortality, major bleeding, and thrombosis is shown in Figure 5. Increased donor exposure was associated with major bleeding or thrombotic events; however, no difference in mortality was observed.

**FIGURE 5:**
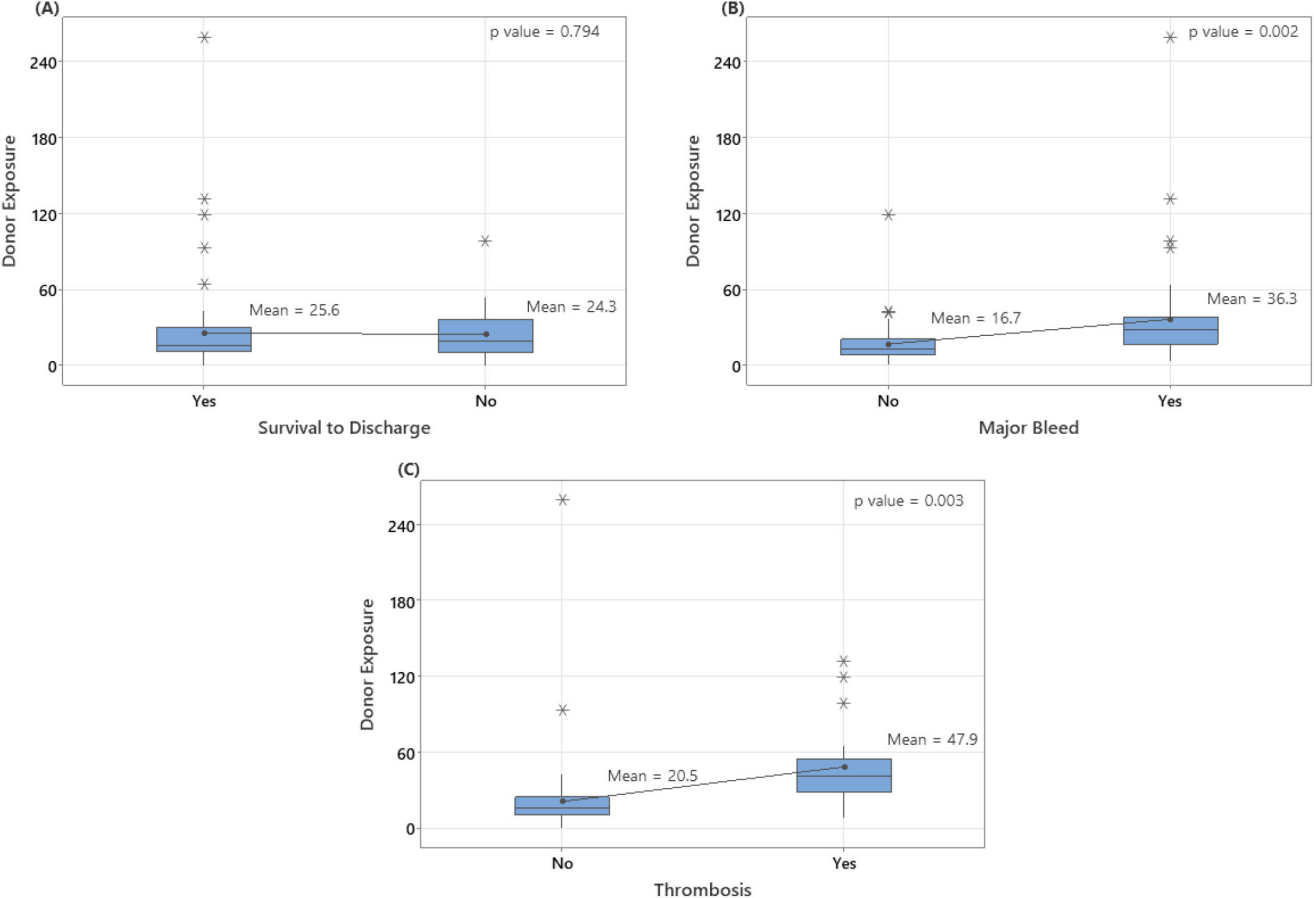
Panels A–C illustrate the relationship between total donor exposures and clinical outcomes of mortality, major bleeding, and thrombosis. Panel A: survival to discharge; B: major bleed; C: thrombosis.

Multivariate analysis failed to show any relationship with respect to outcomes of mortality, major bleeding, or thrombosis when compared to daily transfused volume of blood products (RBCs, PLT, Cryo, or FFP), donor exposure, age at time of commencement, primary ECMO indication, type of cannulation, mode of ECMO, or Post‐CPB.

## 4 DISCUSSION

This prospective study describes in detail the blood product utilization (including priming of circuits vs. other transfusions) of neonatal and pediatric ECMO patients and its correlation with clinical outcomes of bleeding, thrombosis and mortality. RBC primes are frequently utilized in young children on ECMO, the inclusion of this volume in the calculation of transfusion volumes can falsely elevate the total RBC requirements, particularly in the case of patients requiring multiple circuit changes.

Previous research suggested that transfusion of blood products is an independent prognostic factor for increased mortality in adult and pediatric studies. This study failed to demonstrate that (with both the exclusion and inclusion of the ECMO prime) the volume of red blood cells, FFP and platelets was predictive of increased mortality. Increased volume of platelets correlated with those patients who bled, but not thrombosis or mortality. Importantly, there was no relationship between overall donor exposure and mortality, although donor exposure correlated with increased bleeding and thrombosis outcomes. As expected, the patients who had prior CPB, or those who were cannulated centrally, required higher transfusion volumes of all blood products except for platelets, compared with those who did not, even when the prime was removed, suggesting that surgical intervention is one of the largest predictors of transfusion volumes.

The majority of transfusions in this study occurred during the first 24 h of ECMO, and this likely represents the initial changes in cardiovascular status, intravascular volume, and associated trauma from surgical procedures at the initiation of ECMO. This study is unique in that red blood cells that were used to prime either CPB, ECMO, or CVVH circuits were excluded from further data analysis, hence the red blood cells that were then transfused to the patient were due to clinical conditions or underlying patient pathology, hypoxia or hypoxemia, need for volume expansion or due to laboratory parameters. Of particular importance is the lack of increased volume of red blood cells transfused to patients who experienced major bleeding events and those who did not, suggesting that the trigger for transfusions may not be based on the clinical status of bleeding and resultant Hb in isolation. This observation might also indicate that in children, where the volume of the circuit is at times much greater than the blood volume of the patient, the amount of blood transfused to initiate and stabilize the patient on ECMO makes any other additional transfusion insignificant. In this patient population, iatrogenic causes; such as repeated phlebotomy and surgical procedures, may reduce the impact of other primary pathological causes such as gastrointestinal blood loss, coagulopathies, hemolysis, impaired production and reduced red blood cell survival on total transfusion requirements.^16^, ^17^, ^18^, ^19^, ^20^

Several adult studies have demonstrated that in a variety of clinical settings (both ECMO and without) a restrictive transfusion strategy was as effective, or superior to a liberal transfusion strategy with regards to mortality and clinical outcomes.^10^, ^21^, ^22^, ^23^, ^24^, ^25^, ^26^, ^27^, ^28^, ^29^ Pediatric and neonatal intensive care units have also seen an increase in the use of restrictive transfusion thresholds, with both retrospective observational studies and randomized control trials suggesting a link between transfusion of blood products and an increase in mortality.^4^, ^5^, ^27^, ^30^, ^31^ Two systematic reviews and meta‐analysis published by first by Keir and colleagues looking at red blood cell transfusion in neonates, failed to demonstrate a statistically significant difference, however when Deng and colleagues examined post‐cardiac patients they demonstrated a non‐inferiority when using a restrictive transfusion policy.^32^, ^33^

Specific neonatal and pediatric ECMO studies are limited, with a summary of current evidence shown in Table 3. The majority of studies are retrospective, with either a focus on a specific population group such as neonates with respiratory failure, or a single blood product transfusion. Those that are prospective are often multi‐center, with significant variation in transfusion practices, incidence and management of clinical events, as well as lack of standardized or a priori definitions across the centers.

**TABLE 3:** Summary of current neonatal and pediatric ECMO studies with a focus on blood product transfusion and clinical events.

Authors	Study type	ECMO population	Conclusion	Comparison to current study
Nellis et al.^34^	Subgroup analysis of 2 observational point‐prevalence studies PlasmaTV^a^ (2014) and P^3^T^b^ (2016–12,017) Multicenter (51 PICUs in 13 countries)	Neonatal and pediatric ECMO	Description of plasma and platelet transfusions of children on ECMO.	Multiple institutions with minimal overlap in time course. Stated variation between centers with regards to liberal or restrictive transfusion thresholds. Transfusions were for the total PICU admission and not just while on ECMO. Data on mode of ECMO, type of cannulation, indication for ECMO or duration of ECMO was not collected.
Jackson et al.^35^	Retrospective review Extended timeframe (1984–2011)	Neonates ONLY Non‐cardiac	Leukocyte reduced RBCs reduces mortality.	Single age group and limited diagnostic indications: neonatal and non‐cardiac. –Cardiac patients comprise our biggest subpopulation. Leukocyte reduced products are the only products now available in Australia and many international ECMO centers. The conclusion was based on leukoreduction of RBCs and not the transfusion itself. Extended timeframe likely reflects change in ECMO circuits and practices.
Sawyer et al.^36^	Retrospective cohort study Single center (2009–2014)	Neonates ONLY Respiratory failure	Lower target threshold of Hct is associated with reduction in RBC volume. No change in complications between cohorts.	Single patient diagnostic group only: neonatal respiratory. – Cardiac patients comprise our biggest subpopulation. Excluded blood products for priming of the ECMO circuit Thrombotic and hemorrhagic complications extracted from ELSO registry. First ECMO circuit ONLY. Transfusion thresholds other than Hct not discussed, that is, platelet count or coagulation assays, or intensivist directed.
Duarte et al.^37^	Retrospective study Single center (2010–2019)	Neonatal and pediatric ECMO No surgical patients	Restrictive policy on ECMO is safe with regards to platelets and RBCs.	No specific guidelines for transfusion targets, however general guidelines suggest a restrictive strategy. Excluded patients who required major surgery during ECMO support. Excluded blood products for priming of the ECMO circuit. Thrombosis was not included. Bleeding definition: “active bleeding” and all cases both mild and major were reported. Relatively low volume single center with 53 patients over 10 years, likely reflects change in ECMO circuits and practice. Platelet and RBCs transfusions only.
Fisher et al.^38^	Retrospective observational study Single center (2010)	Neonatal and pediatric ECMO	RBC transfusion (including donor numbers) was compared with tissue oxygenation and showed no measurable benefit.	No clinical outcomes investigated. No guidelines, laboratory or clinical thresholds to guide transfusion. RBC transfusions only. Bleeding definition: listed as mL/kg and not bleeding site or categorized into severity.
Gancar et al.^6^	Retrospective review Single center Extended timeframe (2002–2019)	Neonates ONLY Respiratory failure	RBC is associated with mortality but not platelets or plasma	Majority were VV ECMO. Guidelines for transfusion of RBC, platelets and cryoprecipitate only and NOT FFP. Routine replacement of antithrombin III (ATIII). Cryoprecipitate and FFP volumes were considered together. Targeted Hct. Bleeding and thrombosis definition: not clearly defined or a priori.
Dalton et al.^19^	Prospective observational Multicenter (eight centers) (2012–2014)	Neonatal and pediatric ECMO	Bleeding and thrombosis during ECMO (BATE)	Multicenter study with authors stating significant variation in event rates (bleeding and thrombosis) between centers. Bleeding definition: intracranial bleeding and requiring transfusion. – Subjective bleeding definition. Thrombotic definition: list of classification, subjective and not specific.
Muszynski et al.^7^	Prospective observational Multicenter (eight centers) (2012–2014)	Neonatal and pediatric ECMO	Bleeding and thrombosis during ECMO (BATE) – RBCs ONLY Transfusion volume was associated with mortality	Multicenter study with authors stating significant variation in transfusion practice across centers. Bleeding definition: requiring transfusion Thrombosis not discussed. No information regarding nature of products such as leukodepleted, irradiation and storage. Whole blood and packed RBCs transfused. Targeted Hct.
Cashen et al.^8^	Prospective observational Multicenter (eight centers) (2012–2014)	Neonatal and pediatric ECMO	Bleeding and thrombosis during ECMO (BATE) Platelet volume—increased mortality, bleeding and thrombosis	Multicenter study with authors stating significant variation in transfusion practice across centers. Indications for transfusion were not recorded. Bleeding definition: requiring transfusion. Thrombotic definition: list of classification and not specific.
Keene et al.^3^	Retrospective review Single center (2010–2016)	Neonates ONLY Respiratory failure	RBC and platelet transfusion rates are associated with in‐hospital mortality	Data from the first 24 h following ECMO cannulation was not considered as part of the primary outcome. Mortality and no other clinical outcomes were investigated for association. Excluded ECMO prime. Bleeding and thrombosis definition: not clearly defined or a priori. Guidelines for transfusion of RBC, platelets and cryoprecipitate only were available but compliance not reviewed. Targeted Hct.
Smith et al.^9^	Retrospective review Single center 10 year period	Neonatal and pediatric ECMO	Increased RBC volumes in non‐cardiac indications are associated with increased odd of mortality.	Transfusion thresholds and product volumes were at discretion of intensivist. Unclear if guideline based. Bleeding and thrombosis definition: subjective and not a priori RBC and platelet data only. Variation in hemostatic strategies over the decade, such as aprotinin, anti‐fibrinolytics and activated factor VII.

Abbreviations: Hct, hematocrit; RBCs, red blood cells.

^a^ PTV = plasma transfusion value.^39^

^b^ P^3^T = point prevalence of platelet transfusions in critically ill children.^40^

What is unclear from this and other studies, and an important factor in ECMO, is whether the mortality reflects the underlying disease severity, the effect of increasing fluid volume and subsequent pulmonary edema, or is related to the use of anticoagulants, the transfusion of blood products and resultant immune response. Although a restrictive strategy continues to be popular, it should be highlighted that transfusion policies vary widely among clinicians and institutions.^41^, ^42^ The previous ELSO anticoagulation guidelines published in 2017, as well as those in the “Red Book” recommend aiming for a normal Hb, despite there being no specific evidence base to support either a restrictive or liberal strategy.^43^ The more recent guideline from 2021 does not formally recommend a new strategy but suggests a possible example of a more restrictive policy, which includes separation into bleeding and non‐bleeding patients.^42^ They do not define a safe lower Hb threshold but suggest that clinicians focus on markers of adequate regional and systemic oxygen delivery in addition to the actual Hb value, which reinforces the constant aim of ECMO, which is to maintain tissue oxygenation.

Current research has focused on tissue oxygenation, such as cerebral regional tissue oxygenation (crSO2), as a measure of transfusion efficacy, with some studies demonstrating a statistically significant increase following red blood cell transfusion.^44^ The effect was maximal in pediatric ECMO patients who had the lowest crSO2 pretransfusion, however showed no difference between the various pre‐transfusion hemoglobin subgroups (<10, 10–12, >12 g/dL). Similar results from Fiser et al. reported that only a minority (9%) of red blood cell transfusions altered crSO2.^38^ Although not reported in either study, previous data has found that a decrease in crSO2 during ECMO is associated with worse short‐term neurological outcomes, in both pediatric and adult patients.^44^, ^45^, ^46^ Further research is needed to focus on target organ oxygenation, specific indications for transfusion of blood products, preparation and storage of products, and possible transfusion reactions.

The rates of bleeding and thrombosis (35% and 8% respectively) from our study are comparable to those from other ECMO centers and the ELSO registry report, with the accepted limitation that there is a widespread lack of detail and standardization with regards to classification of events.^47^, ^48^ All patients were managed with protocol‐based administration of unfractionated heparin (UFH), targeting a combination of activated clotting time (ACT), and activated partial thromboplastin time (APTT) levels. Given the variation in underlying patient pathophysiology, indication for ECMO including cardiac surgery prior, as well as duration of ECMO it is difficult to define specific cofounders for bleeding and thrombosis.

This study is limited by the sample size, with 110 runs of ECMO, across 100 patients at a single center, and therefore representative of specific transfusion protocols, product specifics and anticoagulation protocols. Specific indications for each transfusion were not available for analysis, with acknowledgement that despite protocols and procedures there is likely to be clinician variation. In addition, transfusion reactions and long‐term blood transfusion complications after ECMO decannulation were not examined. This study does however provide a comprehensive description and analysis of blood product exposure within this complex and critically unwell cohort of pediatric patients.

Regardless, this prospective observational studies of ECMO in children, describes comprehensive transfusion data for all enrolled patients. Although measures of tissue oxygenation were not included in this study it provides future goals for examining transfusion effects and possible future clinical outcomes. This study failed to demonstrate that the volume of red blood cells, FFP, cryoprecipitate and platelets, or total donor exposure was predictive of increased mortality.

## AUTHOR CONTRIBUTIONS

Dr. Barton, Dr. Yaw and Ms. Van Den Helm were involved in study set up, ethics and patient recruitment. Dr. Barton, Dr. Monagle, Ms. Letunica, Ms. Karlaftis and Mr. Cai were involved in data collection, database set up and analysis. Drs. Barton, Monagle, Attard, Maclaren, Ignjatovic and Butt were responsible for overall study design, monitoring and analysis. Dr. Barton analyzed the data and drafted the article under the supervision of Dr Monagle. All authors contributed to revision of the manuscript and approval of the final version.

## FUNDING INFORMATION

Supported, in part, by a grant from Australian National Health and Medical Research Council, grant 1129317.

## CONFLICT OF INTEREST STATEMENT

Dr. Barton received a Postgraduate Health Research Scholarship (PHRS) from the Murdoch Children's Research Institute, Australia. Ms. Van Den Helm's, Ms. Letunica's, and Drs. Ignjatovic's and Monagle's institutions received funding from the Australian National Health and Medical Research Council (grant APP1129317). Dr. MacLaren disclosed that he serves on the Board of Directors of the Extracorporeal Life Support Organization. The remaining authors have disclosed that they do not have any potential conflicts of interest.

## CONSENT

Drs. Barton, Chiletti, d'Udekem, and Ms. Johansen, Mr. Best, and Ms. McKittrick obtained informed consent from participant's parents/guardians for the study.

## Supporting information


**FIGURE S1.** Duration of ECMO run (days) grouped into primary ECMO indication (cardiac, other or respiratory).

**FIGURE S2.** Illustrates the relationship between daily volumes of RBC transfusion and the duration of ECMO with respect to primary indication for ECMO.

## Data Availability

The data that support the findings of this study are available on request from the corresponding author. The data are not publicly available due to privacy or ethical restrictions.
