# Supplementary material for: Transfusion of blood products to neonatal and pediatric extracorporeal membrane oxygenation patients: A prospective, observational study
Source: Transfusion. 2026 Jun 18;66(11):2101–15. doi: 10.1111/trf.70154 (PMC13753056; doi:10.1111/trf.70154)
Supplement: Supplementary file 1 [file TRF-66-2101-s001.pdf]

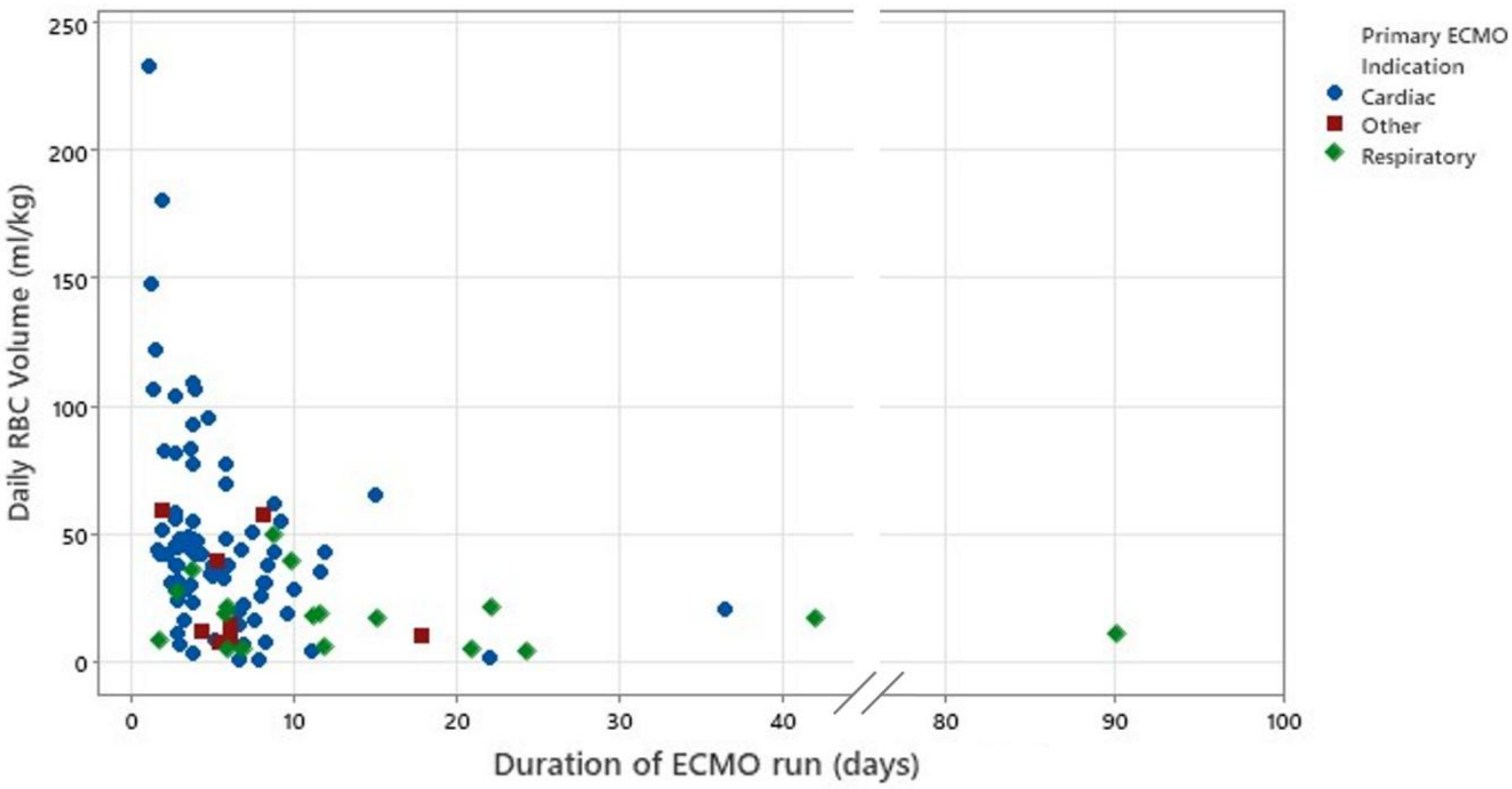

Daily RBC Volume (ml/kg)
250
200
150
100
50
0
0
10
20
30
40
80
90
100
Duration of ECMO run (days)
Primary ECMO Indication
Cardiac
Other
Respiratory
