# Supplementary material for: Transfusion of blood products to neonatal and pediatric extracorporeal membrane oxygenation patients: A prospective, observational study
Source: Transfusion. 2026 Jun 18;66(11):2101–15. doi: 10.1111/trf.70154 (PMC13753056; doi:10.1111/trf.70154)
Supplement: Supplementary file 2 [file TRF-66-2101-s002.pdf]

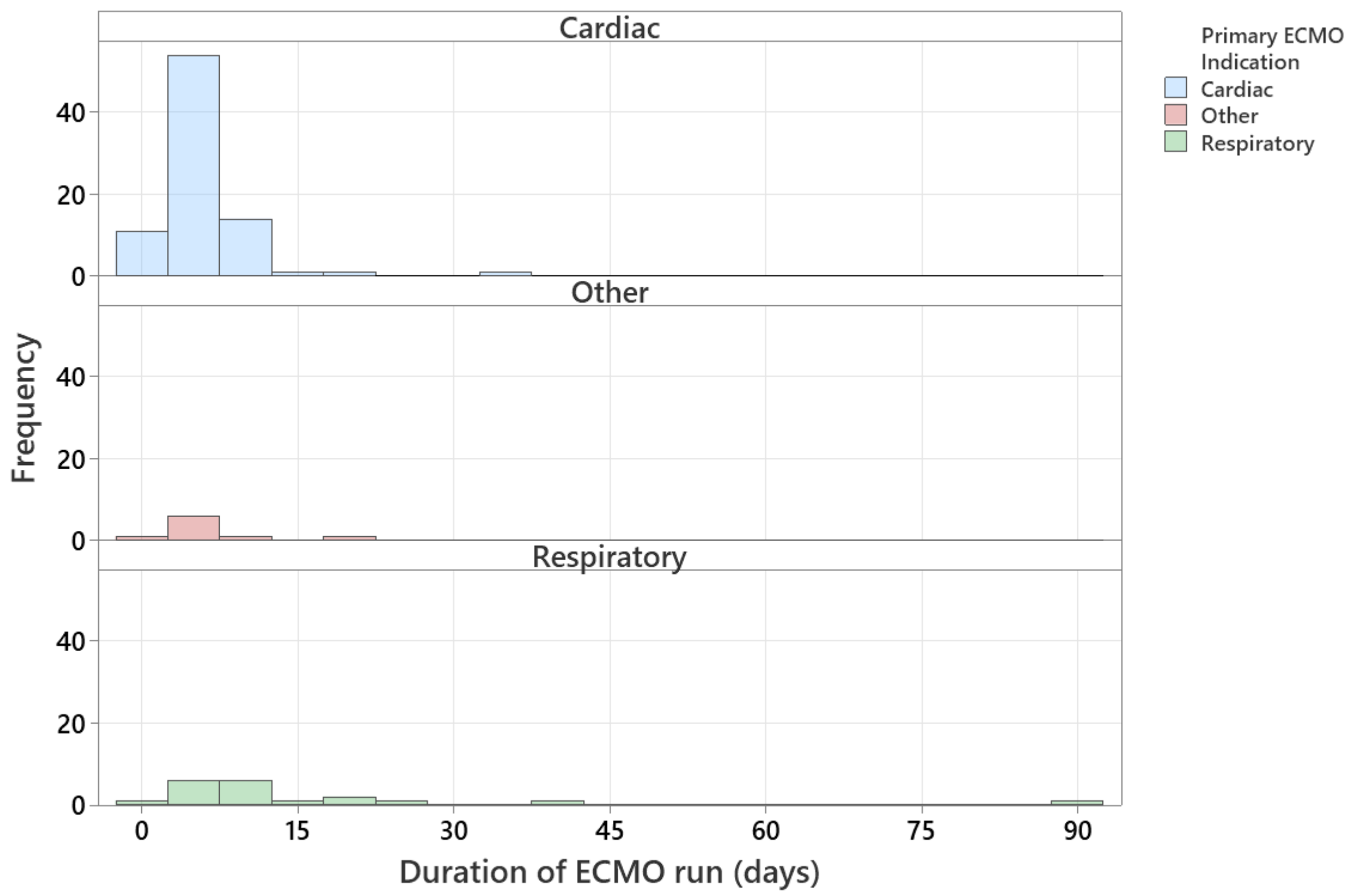

Panel variable: Primary ECMO Indication
